# Maintenance of Hepatic Functions in Primary Human Hepatocytes Cultured on Xeno-Free and Chemical Defined Human Recombinant Laminins

**DOI:** 10.1371/journal.pone.0161383

**Published:** 2016-09-06

**Authors:** Masaaki Watanabe, Helen Zemack, Helene Johansson, Louise Hagbard, Carl Jorns, Meng Li, Ewa Ellis

**Affiliations:** 1 Department of Clinical Science, Intervention and Technology (CLINTEC), Division of Transplantation Surgery, Karolinska Institutet, Stockholm, Sweden; 2 BioLamina AB, Stockholm, Sweden; Georgetown University, UNITED STATES

## Abstract

Refined methods for maintaining specific functions of isolated hepatocytes under xeno-free and chemical defined conditions is of great importance for the development of hepatocyte research and regenerative therapy. Laminins, a large family of heterotrimeric basement membrane adhesion proteins, are highly cell and tissue type specific components of the extracellular matrix and strongly influence the behavior and function of associated cells and/or tissues. However, detailed biological functions of many laminin isoforms are still to be evaluated. In this study, we determined the distribution of laminin isoforms in human liver tissue and isolated primary human hepatocytes by western blot analysis, and investigated the efficacy of different human recombinant laminin isoforms on hepatic functions during culture. Protein expressions of laminin-chain α2, α3, α4, β1, β3, γ1, and γ2 were detected in both isolated human hepatocytes and liver tissue. No α1 and α5 expression could be detected in liver tissue or hepatocytes. Hepatocytes were isolated from five different individual livers, and cultured on human recombinant laminin isoforms -111, -211, -221, -332, -411, -421, -511, and -521 (Biolamina AB), matrigel (extracted from Engelbreth-Holm-Swarm sarcoma), or collagen type IV (Collagen). Hepatocytes cultured on laminin showed characteristic hexagonal shape in a flat cell monolayer. Viability, double stranded DNA concentration, and Ki67 expression for hepatocytes cultured for six days on laminin were comparable to those cultured on EHS and Collagen. Hepatocytes cultured on laminin also displayed production of human albumin, alpha-1-antitrypsin, bile acids, and gene expression of liver-enriched factors, such as hepatocyte nuclear factor 4 alpha, glucose-6-phosphate, cytochrome P450 3A4, and multidrug resistance-associated protein 2. We conclude that all forms of human recombinant laminin tested maintain cell viability and liver-specific functions of primary human hepatocytes, and that recombinant laminin is a promising xeno-free and chemical defined strategy for preservation of hepatocyte specific function *in vitro*.

## Introduction

During the cell isolating process, hepatocytes are separated from each other and deprived of their original environment [1]. The disruption of cell-to-cell and cell-to-matrix contacts leads to subsequent hepatocyte dysfunction and/or apoptosis, and the isolated hepatocytes lose their specific functions if the cells are not anchored and cultured under appropriate conditions [2–5]. The extracellular matrix contains different specific proteins, such as laminins, type IV collagens, perlecan, and nidogens, forming complex networks that strongly influence the behavior and functions of the associated cells [6]. To maintain specific functions in a culture system, isolated hepatocytes need to be cultured with an extracellular matrix component, usually Matrigel^™^ or collagen. However, due to diversity of biological material, the effect on the cells differs with different matrices. The quality may also differ from batch to batch, making reproducibility difficult. Furthermore, clinical application under good manufacturing practice (GMP) protocols is difficult due to their animal origin. A robust and new strategy for culturing isolated hepatocytes under xeno-free and chemical defined conditions is important for future use of primary and/or stem cell derived hepatocytes in GMP settings.

Laminins are a large family of conserved, heterotrimeric basement membrane adhesion proteins that consisting of α-, β-, and γ-chains. Laminins contribute to the structure of extracellular matrix and influence the behavior of associated cells, such as adhesion, migration, differentiation, viability and phenotypic stability [7]. In vertebrates, there are at least 16 different laminin isoforms present, and they are named based on their chain composition, for example, the composition of laminin-111 is α1β1γ1 [8]. Previous studies have shown that human liver express laminins [9, 10] and rat hepatocytes cultured on laminins maintain expression of hepatic differentiation markers, such as tyrosine aminotransferase, tryptophan-2, 3-dioxygenase, and cytochrome P450 [11]. Identification of the laminin isoforms maintaining viability and specific functions of human hepatocytes would represent a further advancement.

In the current study, we determined the expressions of laminin chains in isolated primary human hepatocytes and liver tissue, and investigated the efficacy of human recombinant laminins on hepatocyte functions *in vitro*, such as albumin production, hepatic transport activity, and hepatic metabolism.

## Materials and Methods

### Isolation and culture of human hepatocytes

Liver tissue was obtained from five patients undergoing liver resection, liver transplantation, or from deceased organ donors (one female, four males). Donor age ranged from 14 to 74 years (Table 1). Informed written consent was obtained from all patients or from next of kin on behalf of children and documented in the patient medical file. The Regional Ethics Committee, Stockholm, Sweden, approved the study including the consent procedure (Dnr: 2010/678-31/3). Primary human liver tissue from deceased liver donors were included in the study according to the regulations of the organ transplantation law of Sweden (1995:831), that is, the donor’s prior written declaration was followed as well as the written informed consent. Hepatocytes were isolated using a standardized two-step collagenase perfusion technique as previously described [1, 12]. Briefly, silicone catheters were sutured into major hepatic vessels on the cut surface of the tissue. Catheters were connected to a peristaltic pump and the liver specimen placed in a sterile bag in a water bath at 37°C. The liver specimen was perfused at 10–200 ml/min depending on the size of the tissue. The first perfusion solution consisted of Hank’s Balanced Salt Solution (HBSS; without calcium, magnesium and phenol red; Gibco, Life Technologies, Inc., Rockville, MD, USA) supplemented with 0.5 mmol/L ethylene glycol tetraacetic acid (EGTA). After a washout with EGTA free HBSS, the tissue was perfused for 20–30 min with a third solution composed of Eagle's Minimum Essential Medium (Lonza, Basel, Switzerland) supplemented with 250 mg/L collagenase Type XI (Sigma*-*Aldrich, St. Louis, MO, USA) and 50 mg/L DNase (Sigma-Aldrich). Tissue was perfused with solution three for 20–30 min while the solution was recirculated. Digested tissue was then mechanically disrupted in ice cold Eagle's minimal essential medium (EMEM). Hepatocytes were pelleted by centrifugation at 70g for 5 min at 4°C. The final hepatocyte pellet was re-suspended in Williams E medium (Sigma-Aldrich). Cell number and viability was assessed by standard Trypan blue (Sigma-Aldrich) exclusion test.

**Table 1:** Characteristics of hepatocytes donors.

	Age	Gender	Diagnosis	Viability of isolated hepatocytes (%)
**# 1**	65	male	Liver metastatic tumor of colon carcinoma	92.6
**# 2**	44	male	Deceased donor	75.0
**# 3**	74	female	Hepatocellular carcinoma	90.0
**# 4**	14	male	Crigler-Najjar syndrome	76.0
**# 5**	53	male	Deceased donor	70.0

Hepatocytes were isolated from 5 different individuals (one female, four males). Liver tissue was obtained from patients undergoing liver resection, liver transplantation, or from deceased organ donors. Donor age ranged from 14 to 74 years.

### Reagents

Human recombinant laminin isoforms -111, -211, -221, -332, -441, -421, -511, and -521 (BioLamina AB, Stockholm, Sweden), matrigel extracted from Engelbreth-Holm-Swarm murine sarcoma (EHS; Corning), and collagen type IV (Collagen; Sigma*-*Aldrich) were pre-coated on 6, 12, 24, and 96-well plates (BD Biosciences, Mountain View, CA, USA) at least 120 min before seeding.

### Cell culture

Isolated primary human hepatocytes were seeded on plates pre-coated with laminin (10 μg/ml), EHS, or Collagen (0.3 mg/ml) at a density of 7.5 x 10^5^ cells/ml in Williams E medium (Sigma*-*Aldrich) solution supplemented with 100 nM dexamethasone, 2 mM Glutamine (Sigma*-*Aldrich), 12 nM insulin (Novo Nordisk Pharma, Malmö, Sweden), 20 mM 4-(2-hydroxyethyl)-1-piperazineethanesulfonic acid (HEPES), 0.01 M Gentamicin, and 50 nM Amphotericin B (all from Lonza) at 37°C, with 5% CO_2_ and in a humidified atmosphere. The laminin concentration, 10 μg/ml, was selected according to the manufacturer's instructions. The medium was collected and replaced during the experiments every 24 hours thereafter.

### MTT assay

The 3-(4,5-dimethylthiazol-2-yl)-2,5-diphenyltetrazolium bromide tetrazolium (MTT; Sigma*-*Aldrich) solution was added to cells in culture medium at a final concentration of 0.5 mg/ml, and incubated for 30 min. At the end of the incubation period, the medium was removed, and the converted dye was solubilized with isopropanol. Absorbance of the converted dye was measured at a wavelength of 590 nm with background subtraction at 650 nm by using Biotek FLx800^™^ Multi-Detection Microplate Reader operated by Gen5^™^ Data Analysis Software.

### Double stranded DNA quantification

Quant-iT^™^ PicoGreen^®^ double stranded (ds) DNA kit was used according to manufacturer’s instructions (Molecular probes, Invitrogen, Camarillo, CA, USA) to quantify dsDNA. Quant-iT^™^ PicoGreen^®^ in TE buffer was added to 96-well plate and the fluorescence intensity was read on a fluorescent spectrometer (Biotek FLx800^™^ Multi-Detection Microplate Reader) at an excitation wavelength of 485 nm and an emission wave length of 520 nm. A fresh standard curve of lambda DNA was analyzed for each assay and results are expressed as mg/ml.

### Measurement of human albumin and α1-antitrypsin secretion

Conditioned medium was collected at day six after seeding, and stored at -80°C until analysis. Human albumin and human alpha-1-antitrypsin (A1AT) were measured by ELISA kit (Abcam, Cambridge, UK) following protocols supplied by the manufactures. Samples were collected from each culture condition and each sample was measured in triplicate.

### Measurement of bile acids in cell culture medium

Primary bile acids, cholic acid (CA) and chenodeoxycholic acid (CDCA), were analyzed in cell culture medium as previously described [13]. Briefly, an internal standard composed of deuterium-labeled cholic acid and chenodeoxycholic acid were added to 1ml of culture medium collected at day six after seeding. The medium and internal standard, was diluted with 2 mL of aqueous ethanol (1/1, vol/vol) and hydrolyzed at 120°C for 12 hours using potassium hydroxide (1 mol/L). The hydrolyzed mixture was diluted with saline and extracted by basic ether extraction followed by acidic ether extraction. The ether phase was washed with water until neutral. The solvent was evaporated and the residue was methylated with diazomethane. The methylated extract was converted into trimethylsilyl ether derivatives with hexamethyl-silazane and trichlorosilane. Bile acids were measured by gas chromatography/mass spectrometry.

### Western blot analysis

Laminin protein levels in isolated hepatocytes and liver tissue were evaluated by western blot analysis. Isolated hepatocyte and corresponding liver tissue from 6 individuals (Table 2) were homogenized and sonicated in cracking buffer. Forty μg total protein were mixed with Laemmli buffer (to a final loading concentration of 2% SDS, 10% glycerol, 0.002% bromophenol blue, 0.0625 M TrisHCl), supplemented with DTT to a final concentration of 50 mM, and incubated in 95°C for 5 min. Protein samples and HiMarkTM Pre-stained Protein Standard (Thermo Fisher Scientific cat # LC5699) were loaded onto Criterion TGX Precast Gels, 4–20% polyacrylamide (Bio-Rad, Hercules, CA) and run at 200 volts on ice for 2 hours. The proteins were transferred from the gels to PVDF membranes through wet transfer according to manufacturer’s protocol (Bio-Rad). To prevent non-specific background binding of the primary and/or secondary antibodies to the membrane, membranes were blocked in milk-based blocking buffer (5% (w/v) non-fat dried milk in TBS with 0.1% (v/v) Tween20) for 1 hour. The primary antibody was diluted (1:1000) in blocking buffer and incubated with the blocked membranes over-night shaking in 4 degrees Celsius. To remove residual primary antibody, the membranes were washed 3 x 10 minutes in TBST (TBS with 0.1% (v/v) Tween20). The secondary antibody for laminin antibodies, HRP-conjugated Goat Anti-Mouse Immunoglobulins (DAKO cat #P0477), was diluted 1:3000 in blocking buffer and for B-aktin, Amersham ECL Rabbit IgG HRP-linked F(ab’)_2_ fragment from donkey (GE healthcare cat # NA9340V), diluted 1:50000 and incubated with the membranes for 1 hour. To remove residual secondary antibody, the membranes were washed 3 x 10 minutes in TBST. The membranes were incubated with detection reagent (Super Signal^™^ West Dura Extended Duration Substrate, Thermo Fisher Scientific cat # 34075) for 5 minutes. The image was captured using a CCD camera. Expressions of each laminin isoform were normalized to the value of β-actin expression.

**Table 2:** Characteristics of hepatocytes and liver tissue donors for western blot analysis.

	Age	Gender	Diagnosis	Viability of isolated hepatocytes (%)
# 1*	65	male	Liver metastatic tumor of colon carcinoma	92.6
# 2*	44	male	Deceased donor	75.0
**# 3**	64	female	Alcoholic cirrhosis	78.0
**# 4**	68	female	Cholangiocellular carcinoma	73.0
**# 5**	57	male	A1ATdeficiency	70.0
**# 6**	6	female	Hyperoxalosis	94.0

*same donor as in Table 1.

### Real time PCR quantification

Total mRNA was isolated from the cultured cells or liver tissue with Trizol (Ambion Life technologies, Waltham, MA, USA) using the manufactures protocol and dissolved in 50 μl RNase free water. cDNA synthesis from 1μg RNA was performed by using Applied Biosystem’s High capacity cDNA reverse transcription kit. Quantification of mRNA was performed using TaqMan real time PCR, employing the Applied Biosystems Step one plus Real-Time PCR system. Relative mRNA expression was calculated from the Ct-values against the house keeping gene PPIA using the comparative delta-Ct method. The following primers targeting specific mRNA were used: Hs99999904_m1 *(peptidyl-prolyl cis-trans isomerase A; PPIA)*, Hs00910225_m1 *(albumin; Alb)*, Hs01097800_m1 *(alpha-1-antitrypsin; A1AT)*, Hs00230853_m1 *(hepatocyte nuclear factor 4 alpha*; *HNF4a)*, Hs00609178_m1 *(glucose-6-phosphate; G6P)*, Hs00430021_m1 *(cytochrome P450 3A4; CYP3A4)*, Mm00484150_m1 *(cytochrome P450 7A1; CYP7A1)*, Mm01278617_m1 *(Ki67)*, and Hs01385685_m1 *(multidrug resistance-associated protein 2; MRP2)*.

### Statistical analysis

Normal distribution was assessed by skewness (histograms). Due to a low number of samples and skewed data non-parametric Kruskal-Wallis with Dunn’s multiple comparison test to correct for multiple comparisons was used to evaluate differences between samples. A *p*-value of < 0.05 was considered statistically significant and all calculations were performed using GraphPad Prism^®^ software (GraphPad Software Inc., San Diego, CA).

## Results

### Expression of laminin in liver tissue and isolated hepatocytes

**Fig 1:**
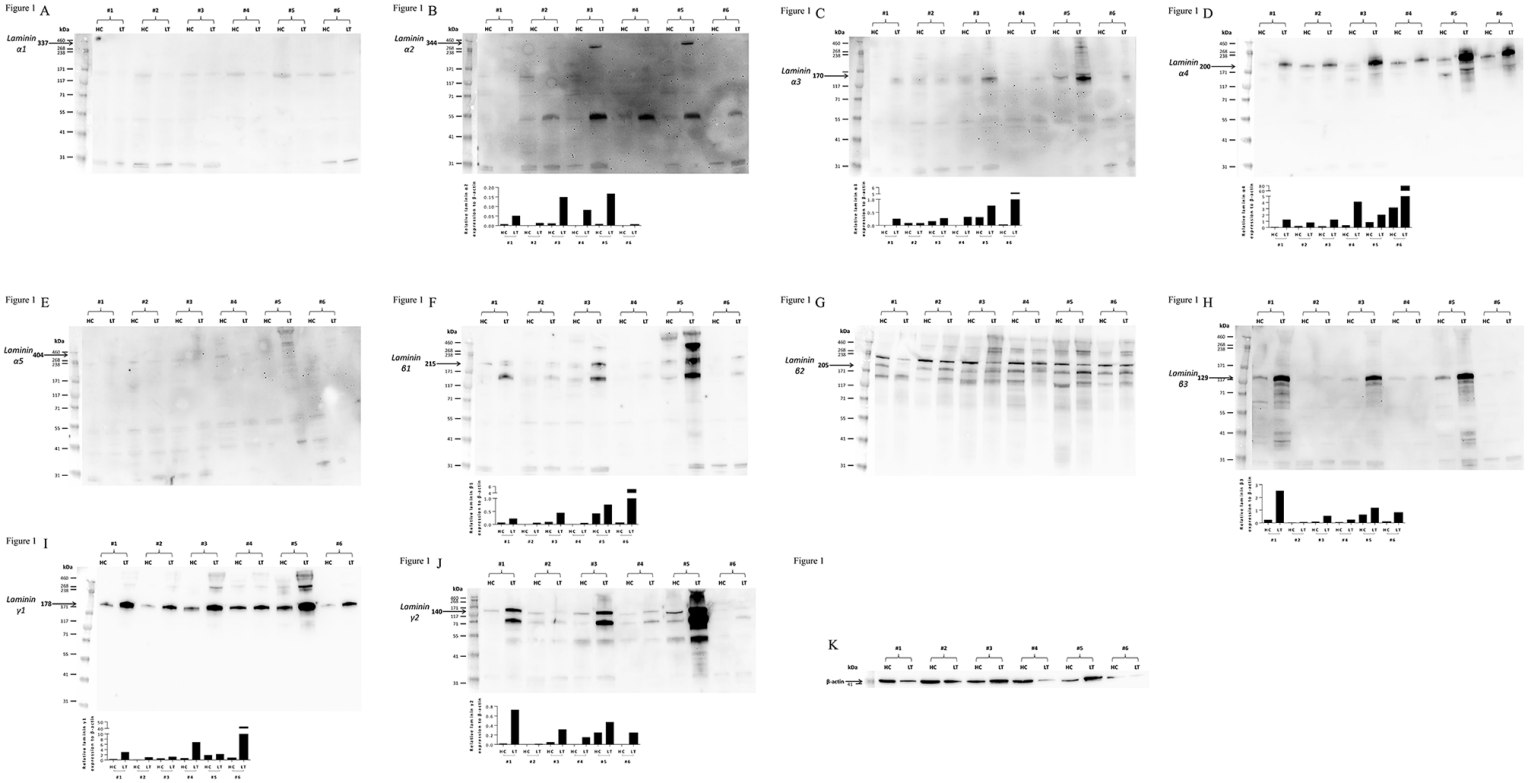
Expression of laminin in human liver and isolated hepatocyte. Expression of laminin was determined in protein samples from liver tissue (LT) and isolated hepatocytes (HC) by western blot analysis. (A; Laminin-α1, B; Laminin-α2, C; Laminin-α3, D; Laminin-α4, E; Laminin-α5, F; Laminin-β1, G; Laminin-β2, H; Laminin-β3, I; Laminin-γ1, J; Laminin-γ2, K; β-actin) Phenotypes of laminins, α2, α3, α4, β1, β3, γ1, and γ2 were detected both in liver tissue and isolated hepatocytes, and α1 and α5 were absent in both. Laminin chain of β2 was inconclusive due to multiple bands. Sample number (#1–#6) is corresponding to Table 2. Values were normalized to the level of β-actin in each lane.

The protein expressions of the different laminin chains in human liver and isolated hepatocytes were evaluated by western blot analysis. Laminin chains of α2, α3, α4, β1, β3, γ1, and γ2 were detected both in liver tissue and isolated hepatocytes, and α1 and α5 were absent in both. Laminin chain of β2 was tested with two different antibodies but was inconclusive due to multiple bands. The expressions of laminin were stronger in liver tissue than in isolated hepatocytes, and there were individual variations between patients (Fig 1).

### Hepatocyte morphology

**Fig 2:**
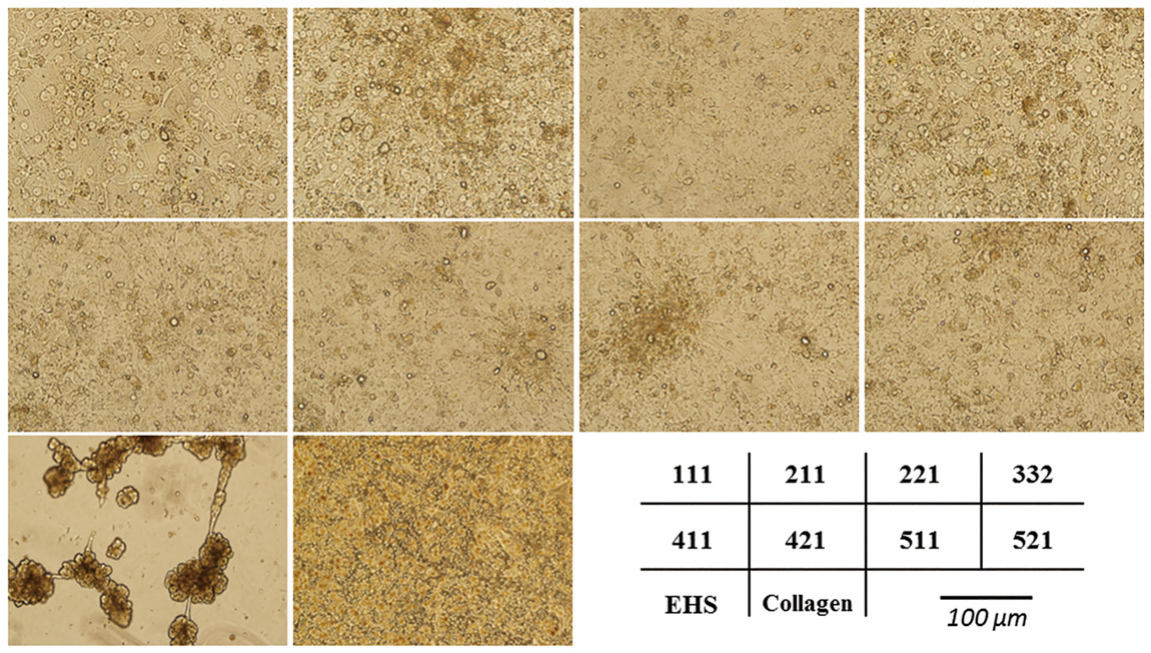
Morphology of primary human hepatocytes seeded on laminin, EHS, or Collagen. Isolated primary human hepatocytes were seeded on laminin isoforms -111, -211, -221, -332, -411, -421, -511, and -521, EHS, or Collagen pre-coated plates. Hepatocytes cultured on EHS coated plates migrated and formed three-dimensional structures. Hepatocytes cultured on laminins and collagen showed a hexagonal shape in a flat cell monolayer. Isolated human hepatocytes could be incubated on laminins for up to six days without obvious morphological changes.

Isolated primary human hepatocytes were cultured on different laminin isoforms -111, -211, -221, -332, -411, -421, -511, and -521, EHS, or Collagen pre-coated plates, and were evaluated morphologically. Hepatocytes cultured on EHS migrated within a few days and formed three-dimensional cell clusters while hepatocytes cultured on laminin or Collagen showed characteristic, hexagonal shape in a flat cell monolayer for up to six days (Fig 2). Morphological differences in shape, density, and appearance of nuclei between different laminin isoforms were seen, however, this was difficult to measure.

### Viability of hepatocytes

**Fig 3:**
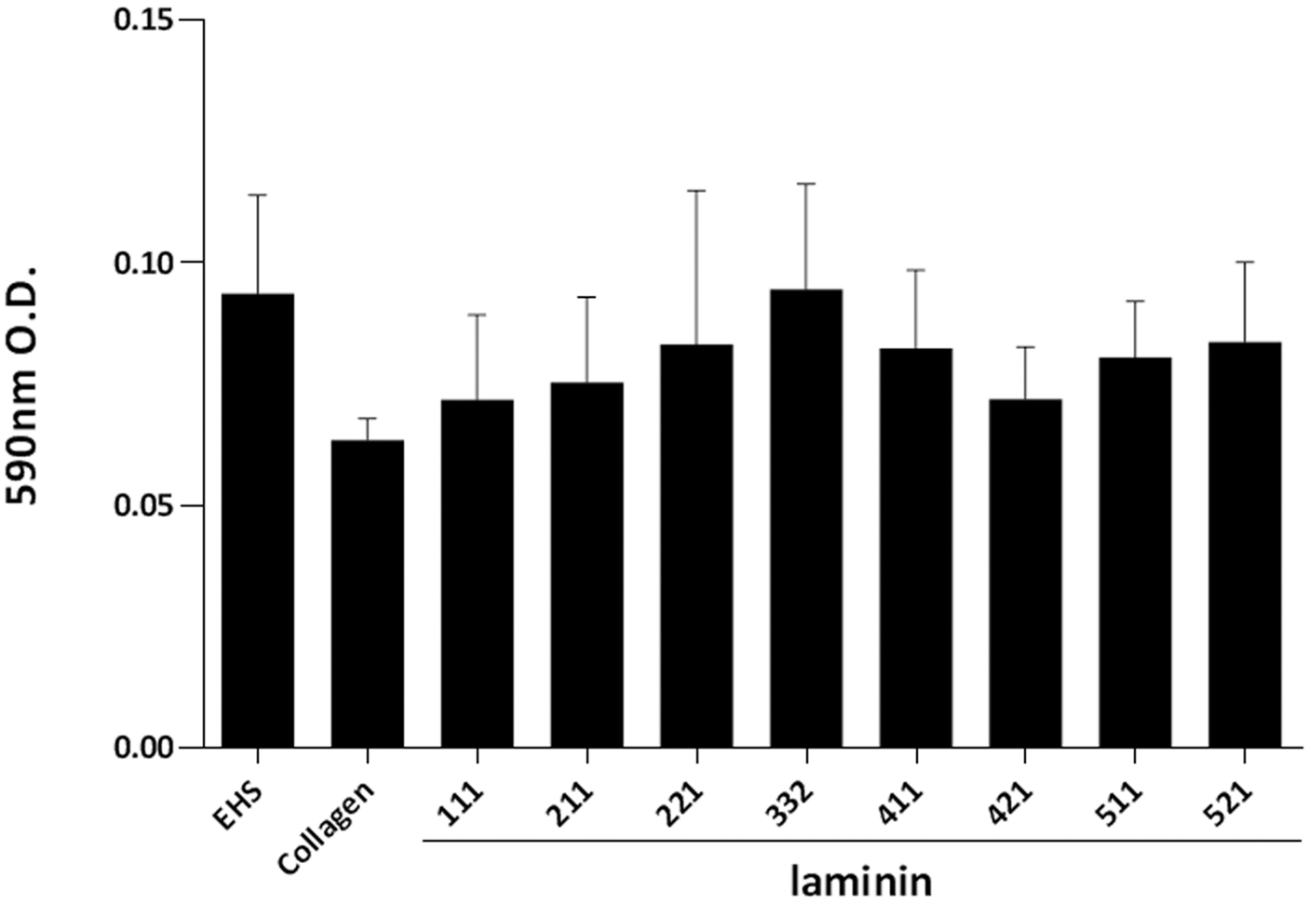
Viability of cultured hepatocytes. Hepatocyte viability during the culture was assessed at day six after seeding by MTT assay. Each laminin isoform supports hepatocyte viability during culture for at least 6 days, and no statistical differences between each laminin, EHS and Collagen were seen. The results are from five replicates and are expressed as mean ± SEM.

Hepatocyte viability was assessed by MTT assay at day six after seeding. All laminin isoforms maintained hepatocyte viability comparable to Collagen and EHS. No statistical differences between the different laminins, EHS and Collagen were seen (Fig 3).

### dsDNA concentrations and mRNA expression of Ki67

**Fig 4:**
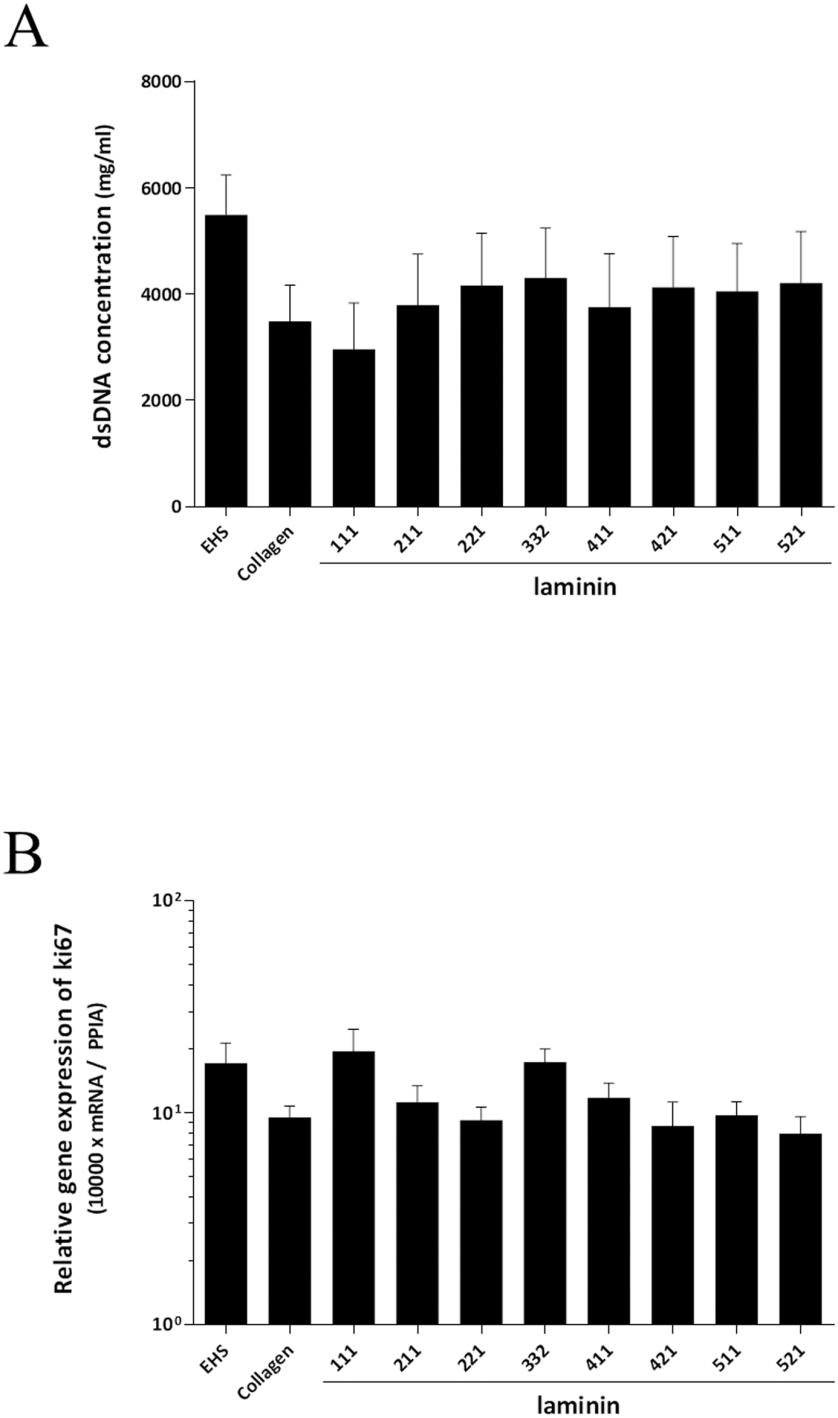
dsDNA concentrations and mRNA expression of Ki67. The dsDNA concentrations (A) and mRNA expression of Ki67 (B) were evaluated at day six after seeding. No significant differences in dsDNA or Ki67 expression could be noted between different laminin isoforms, Collagen and EHS. The results from five replicates are expressed as mean ± SEM.

The dsDNA concentrations (Fig 4A) and mRNA expression of Ki67 (Fig 4B) were evaluated in cultured hepatocytes at day six after seeding. No significant difference in dsDNA concentration or Ki67 expression was seen between the different laminin isoforms and the values were comparable to the values seen for hepatocytes cultured on EHS or Collagen.

### Production of human albumin and A1AT

**Fig 5:**
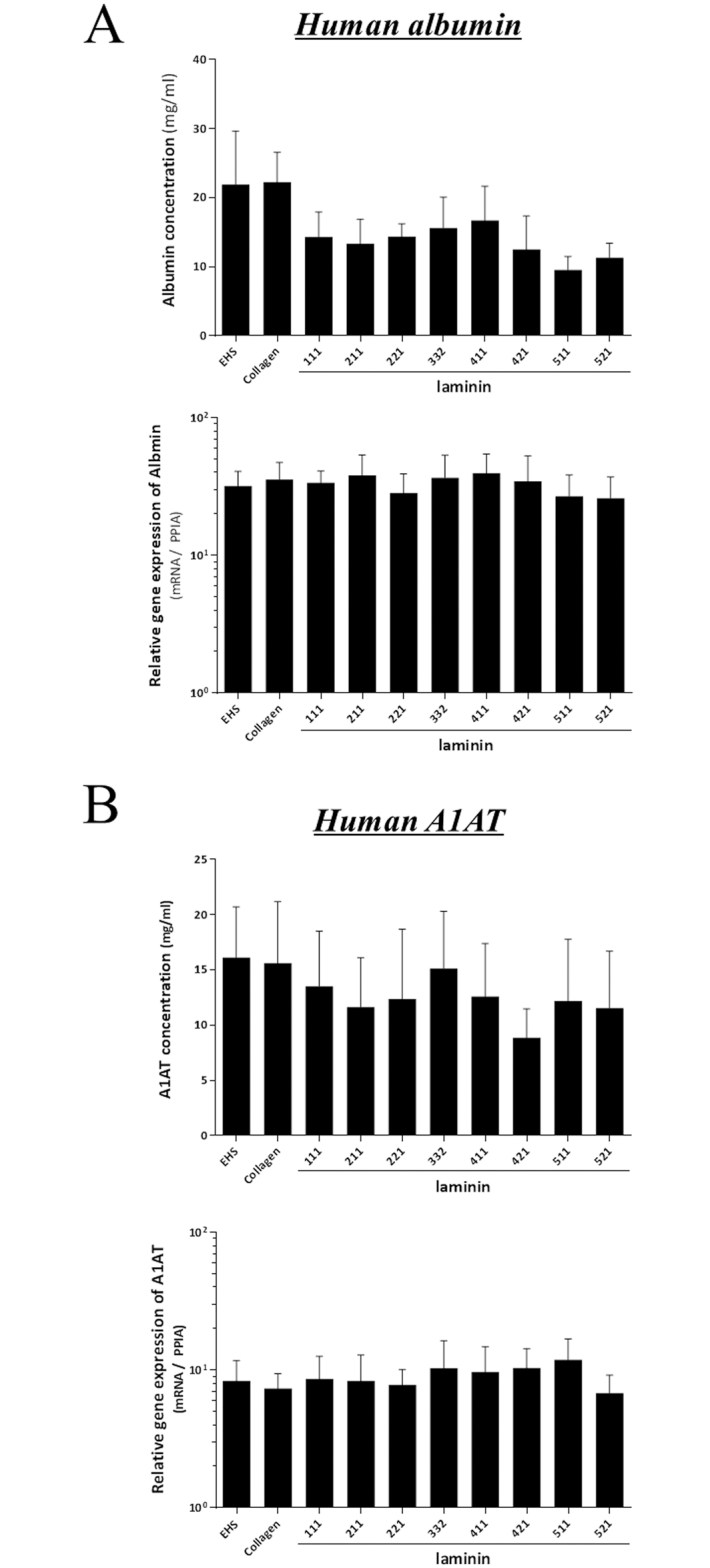
Productions and mRNA expressions of human albumin and A1AT. Production and mRNA expression of human albumin (Fig 5A) and human alpha-1-antitrypsin (A1AT; Fig 5B) were evaluated by ELISA and RT-PCR at six days after seeding. Hepatocytes cultured on laminin maintained the production and mRNA expressions of albumin and A1AT as compared to those of hepatocytes on EHS or Collagen. No significant differences in the productions and mRNA expressions of human albumin and human A1AT could be noted among different laminin isoforms, Collagen and EHS. The results from five replicates are expressed as mean ± SEM.

We evaluated liver functions by human albumin and human A1AT synthesis and mRNA expression. As shown in Fig 5, hepatocytes cultured on laminin maintained mRNA expression and protein secretion of albumin and A1AT equally well compared to those of hepatocytes on EHS or Collagen.

### Bile acid synthesis

**Fig 6:**
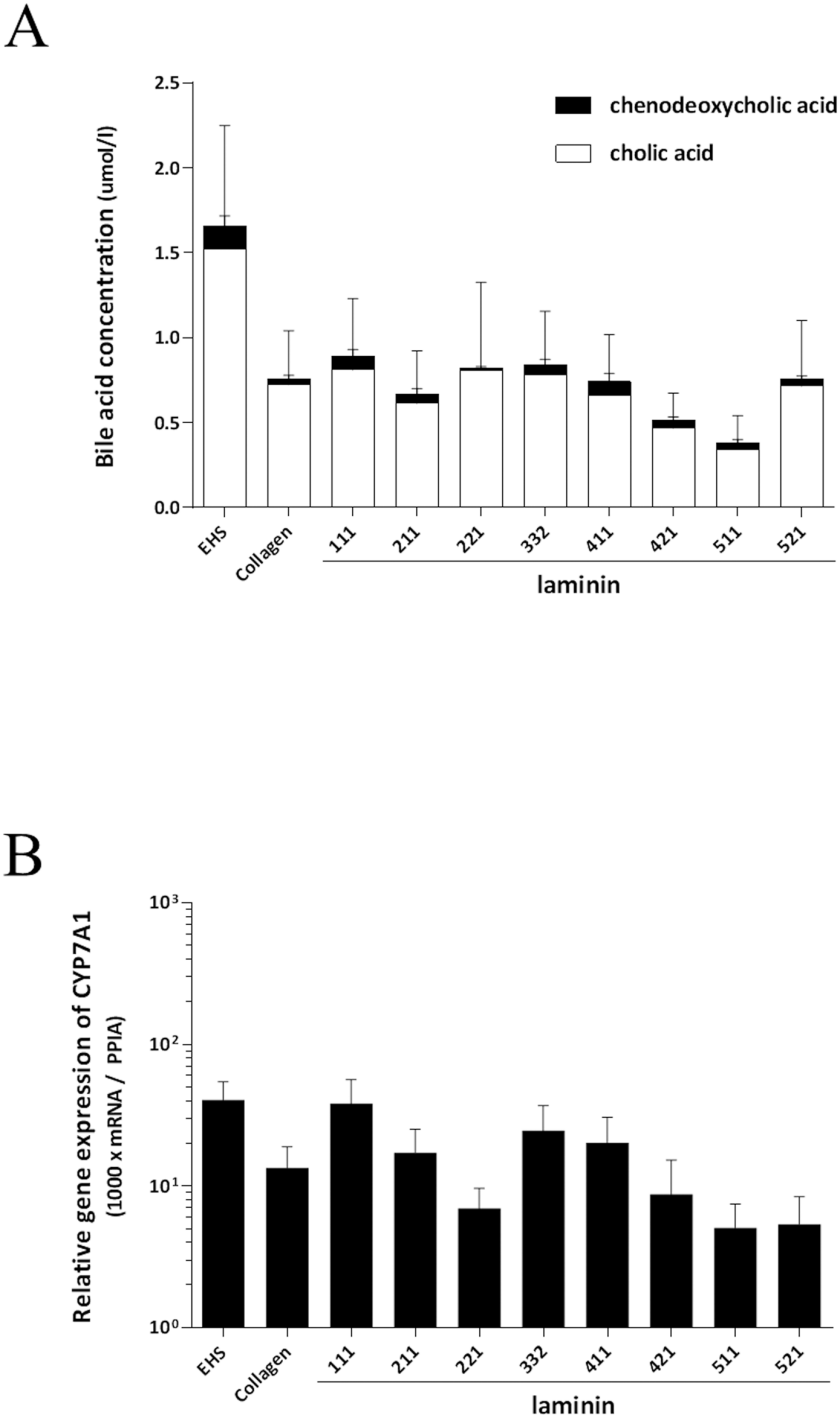
Bile acid synthesis. Bile acid (CA and CDCA) concentrations in the medium were assessed at six days after seeding. All of hepatocytes seeded on laminins, EHS, or Collagen demonstrated almost same ratio of CDCA and CA. In terms of the amount of CA, hepatocytes cultured on EHS produced more compared to cells cultured on laminin-211, -411, -421, -511, and -521, however there was no significant difference in the concentration of CDCA or CA among different laminin isoforms, Collagen and EHS. (A). The mRNA expression of CYP7A1 showed a similar trend of the total amount of CA and CDCA synthesized; hepatocytes cultured on EHS, laminin-111, -332, and -411 showed higher mRNA expression of CYP7A1 (B). There was no significant difference in the mRNA expression of CYP7A1 among different laminin isoforms, Collagen and EHS. The results are from five replicates and are expressed as mean ± SEM.

Bile acid synthesis was assessed as a liver-specific function of primary human hepatocytes. Hepatocytes cultured on laminin showed maintained bile acid production as measured by cholic acid (CA) and chenodeoxycholic acid (CDCA) in medium at day six after seeding. The ratio of CDCA and CA was similar in all groups. There was no significant difference in the production of CDCA or CA among different laminin isoforms, Collagen and EHS (Fig 6A). To assess the bile acid synthesis, mRNA expression of CYP7A1 was also evaluated; CYP7A1 is the rate-limiting enzyme in the first step of classical pathway in bile acid synthesis [14]. The mRNA expression of CYP7A1 showed a similar trend of the total amount of CA and CDCA synthesized; where hepatocytes cultured on EHS, laminin-111, -332, and -411 showed higher mRNA expression of CYP7A1 (Fig 6B).

### Expression of metabolic genes in isolated hepatocyte

**Fig 7:**
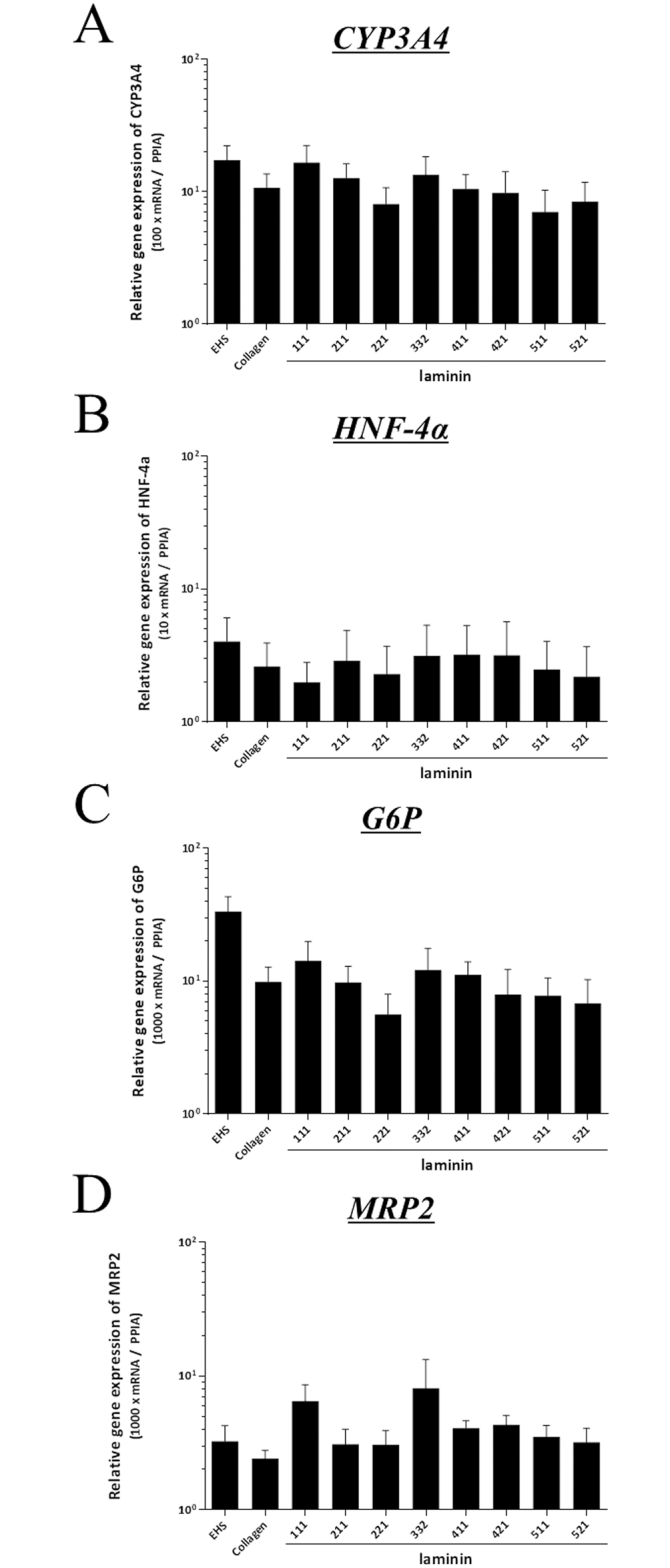
Metabolic activity of isolated hepatocyte. The mRNA expression of CYP3A4 (A), HNF4a (B), G6P (C), and MRP2 (D) of isolated hepatocytes cultured on laminin were assessed. Hepatocytes cultured on laminins demonstrated no significant differences in mRNA expressions of CYP3A4, HNF4a, G6P, or MRP2 as compared to those of Collagen or EHS. The results are from five replicates and are expressed as mean ± SEM.

To evaluate the expression of genes involved in metabolic functions of hepatocytes, mRNA expression of CYP3A4, HNF4a, G6P, and MRP2 of hepatocytes were assessed. Hepatocytes cultured on laminins demonstrated no significant differences in mRNA expressions of CYP3A4, HNF4a, G6P, or MRP2 as compared to those of Collagen or EHS (Fig 7A–7D).

## Discussion

Defined culture system, free of animal components is of great importance for the development of hepatocyte research and regenerative therapy. Conventionally, hepatocytes are often cultured on non-defined matrices, such as EHS (trade name Matrigel^™^). The Matrigel^™^ matrix is of animal origin and consist of a mix of several basement membrane proteins, such as laminin-111, collagen IV, nidogen/enactin and proteoglycan [15] which makes it unsuitable for controlled and defined culture systems. In this study, we evaluated the efficacy of human recombinant laminins as a defined and xeno-free alternative, culture matrix for primary human hepatocytes. We demonstrated that all laminin isoforms tested in this study, -111, -211, -221, -332, -411, -421, -511, and -521, retained viability, gene expressions, and functional properties of primary human hepatocytes for up to 6 days in culture, and of note, these results are comparable to hepatocytes cultured on conventionally used animal derived EHS or Collagen. To our knowledge, this is the first report of culturing and characterizing primary human hepatocytes on human recombinant laminins. Hepatocytes cultured on laminin and Collagen showed characteristic, hexagonal shape in a flat cell monolayer while hepatocytes cultured on EHS rapidly migrated and formed three-dimensional cell cluster. The three-dimensional structure makes it hard to compare the EHS results to the other matrices as three-dimensional culture induces many metabolic functions. An important functional test was to determine whether primary human hepatocytes would maintain the ability to produce bile acids. As shown, hepatocytes cultured on laminin produced cholic acid and chenodeoxycholic acid. The mRNA expression of CYP7A1, the rate-limiting enzyme of the classical pathway in bile acid synthesis, and HNF4a, which regulates CYP7A1 mRNA expression [14], remained unaltered during the culture periods when compared to those of hepatocytes cultured on EHS or Collagen.

Laminins are large trimeric proteins that contain α-chain, β-chain and γ-chain, that can be found in five, three and three genetic variants, respectively. In the human body, at least 16 laminin isoforms with distinct specific roles in cell maintenance adhesion, cell differentiation and migration have been identified [16]. The distribution of laminin chains in human liver tissues is still not fully evaluated but it has been demonstrated that laminin α5 chain is widely observed, except in the sinusoids. The other α chains are variously expressed in Glisson's sheath and central veins. Laminin chains β1, β2, and γ1 chains were also widely observed [9]. In the current study, we evaluated the expression levels of laminin, and found that protein of laminin α2, α3, α4, β1, β3, γ1, and γ2 were detected both in liver tissue and isolated hepatocytes, and α1 and α5 were absent (Fig 1). The discrepancy between the previous study and our current results may be explained by differences in detection methods; immune histochemical analysis or western blot analysis. The intra-individual variability seen in the 6 samples examined in this study also suggests that the discrepancy could be due to variability in patient material and underlying liver diseases that may affect the expression of laminins. Studies on human laminin diseases, such as Pierson syndrome [17] and congenital muscular dystrophy type 1A [18], or gene inactivation in animals [19] have provided knowledge about the detail properties and biological roles of each isoforms of laminin. However, the detailed functions of each laminin-isoforms associated with the liver tissue or isolated hepatocytes remain unclear. Due to co-existence and highly cross-linkage between laminin isoforms [16, 19], partial compensation by others isoforms may mask the specific function. Moreover, different laminin isoforms have common cell adhesion properties, like common integrin binding sites (15). Indeed, in this study, despite no protein expressions of α1, and α5, hepatocytes cultured on isoform of laminin-111, -511, and -521 maintained hepatic functions, such as production of albumin, A1AT, and bile acids and, mRNA expression of CYP3A4, CYP7A1, and G6P.

Laminins also play important roles in many different cellular processes, and laminin-induced cell signaling can influence on gene transcriptions and re-modeling of promoter chromatin leading to proliferation, differentiation and maintenance of different tissues [20]. Pluripotent stem cells derived from the inner cells mass also express laminin chains, and pluripotency of human pluripotent stem cells can be long-term maintained *in vitro* on laminin-521 or -511 without loss of pluripotency and with maintained karyotype [21, 22]. Likewise, it has been increasing recognized that different laminin isoforms are critically important for maintenance and development of different tissues, for example; epithelial cells need laminin-332 together with laminin-511/521, muscle and nerve cells require laminin-211, -221 and -511/521, and endothelial cells grow on laminin-411 in combination with laminin-511 [16]. Hepatoblast-like cells could be maintained long-term when cultured on laminin-111 with the ability to differentiate into both hepatocyte-like cells and cholangiocyte-like cells [23]. The understandings of functional properties of each isoforms of laminin on primary human hepatocytes would provide manageable tools for cell sources of hepatic regenerative therapy, and for hepatocytes transplantation.

From hepatocyte transplants point of view, it has demonstrated that human hepatocytes graft transplanted into the mouse subcutaneous space or under the kidney capsule survived significantly longer-period when extracellular matrix components were provided to the grafts [24]. It has been demonstrated that elevated CYP3A4 activity in hepatocytes grown on matrices or encapsulated to create a 3-dimentional environment [25–27]. The mRNA expression of CYP3A4 in hepatocytes cultured on laminin-111 and -332 in our study could be associated with favorable cell-matrix integrin binding, increased cell-to-cell contact additional secreted extracellular matrix and/or recovery of cell polarity [28, 29]. Given the notion that Matrigel^™^ is rich in laminin-111, and that it is effective for cell attachment and differentiation of hepatocytes *in vitro*, hepatocyte transplantation together with human recombinant laminin would be a promising xeno-free strategy for improvement for the outcome of clinical hepatocyte transplantation. Such studies are currently under progress.

## Conclusions

In summary, primary human hepatocytes cultured on human recombinant laminins showed comparable liver-specific functions compared to those of EHS or collagen. Recombinant laminins offer a xeno-free alternative of long-term culture of primary human hepatocytes allowing its use in hepatocyte regenerative medicine.

## Supporting Information

S1 File Data set of Figs 1, 3, 4, 5, 6 and 7. (XLSX)
